# *Harungana madagascariensis* (Hypericaceae) is a key phorophyte for native epiphytes and lianas during ecological restoration: case study on an oceanic island

**DOI:** 10.7717/peerj.20520

**Published:** 2025-12-19

**Authors:** François M.M.P. Baguette, Cláudia Baider, François Benjamin Vincent Florens

**Affiliations:** 1 Tropical Island Biodiversity, Ecology and Conservation Pole of Research, Department of Biosciences and Ocean Studies, Faculty of Science, University of Mauritius, Le Réduit, Mauritius; 2 The Mauritius Herbarium, Agricultural Services, Ministry of Agro-Industry, Food Security, Blue Economy and Fisheries, Le Réduit, Mauritius

**Keywords:** Biodiversity conservation, Ecological restoration, Ecological succession, Ecosystem dynamic, Facilitation, Heliophyte, Mauritius, Orchids, Restoration policy

## Abstract

Human activities generate multiple pressures on ecosystems, driving rapid biodiversity loss globally. Oceanic islands and tropical forests are most affected by this situation and within them, epiphytes and lianas are among the most threatened floristic components. Yet, they are often understudied and neglected particularly in restoration projects which instead typically favour planting trees and often overlook ecosystem dynamics and functional interactions. We compared native epiphytes and lianas growing on native pioneer trees (*Harungana madagascariensis* (Hypericaceae)) with those growing on other native trees of (1) similar trunk diameter; and (2) similar age, in wet native forests undergoing restoration after invasive alien plant control, on the volcanic oceanic island of Mauritius. We also investigated whether the different phorophytes had any differential influence on the reproductive status of epiphytes and their size. We studied *H. madagascariensis* because it is the dominant native pioneer tree of the island’s wet native vegetation and also because, since decades, it is often controlled by conservation managers. *Harungana madagascariensis* hosted more native epiphyte and liana species than native trees of similar age, but no difference was found with trees of similar diameter. Similarly, there was a higher abundance of epiphyte and liana on *H. madagascariensis* compared to other trees of similar age, but no difference with other trees of similar diameter. Twice more epiphyte/liana species were closely associated with *H. madagascariensis* (multipatt analysis, IndVal 0.31–0.92; *p* < 0.05) compared to other phorophytes of similar diameter, and none were closely associated with other phorophytes of similar age. Finally, *Harungana madagascariensis* hosted more reproductive orchids than phorophytes of similar age and size, but the sizes of epiphytes and lianas did not differ significantly across phorophytes. *Harungana madagascariensis* therefore benefits native epiphytes and lianas, promoting their colonisation after invasive alien plants are controlled, in contrast with other native phorophytes. This contrast is in fact even larger because the cut *H. madagascariensis* are often many meters tall, often already hosting epiphytes, in contrast to seedlings that are planted in their place. On an oceanic island where native biodiversity is acutely threatened and where the benefits of cutting native pioneer trees like *H. madagascariensis* in biodiversity conservation projects remain unproven, our study provides new evidence that the detrimental effects of this management extend beyond the destruction of the native pioneer trees, to also severely hinder the restoration of native epiphyte and liana species. Furthermore, our study underscores how native pioneer trees can help foster the conservation of typically neglected native plant guilds, and the improbable need for stressing that evidence, and not hypotheses, should drive conservation policy.

## Introduction

Global biodiversity is declining rapidly, primarily driven by human activities and their associated impacts (Baillie, Hilton-Taylor & Stuart, 2004; Vie, Hilton-Taylor & Stuart, 2009; Barnosky et al., 2011). In the absence of intensified mitigation efforts, projections indicate that this downward trajectory will persist, potentially resulting in the extinction of up to 12% of species and a 63% reduction in wildlife population densities by the turn of the century (Leclère et al., 2020). Among the different regions of the globe, tropical oceanic islands are particularly impacted by this situation as they host roughly half of the species recognized as threatened in any of the International Union for Conservation of Nature (IUCN) threat categories (IUCN, 2017), including 6,800 angiosperms species estimated to be highly threatened (Caujapé-Castells et al., 2010). Greater efforts of conservation, including of ecological restoration, should therefore be promoted, particularly given that habitat loss remains the most significant driver of biodiversity loss globally (Borges, Gabriel & Fattorini, 2019) and continues despite the creation of protected areas (Mora & Sale, 2011; Hill et al., 2015).

In response to this global biodiversity crisis, governments have committed to several international frameworks, such as the Convention on Biological Diversity (CBD) and the United Nations Sustainable Development Goals (SDGs) (CBD, 2011; United Nations, 2015). In 2022, the UN strengthened its commitment by introducing new targets under the Global Biodiversity Framework (*e.g.*, Target 2), emphasizing the restoration of degraded terrestrial ecosystems (Global Biodiversity Framework (https://www.cbd.int/gbf)). Furthermore, large-scale initiatives like the Bonn Challenge (2011) and the UN Decade on Ecosystem Restoration (2021–2030) have been launched to drive coordinated global action. Overall however, planting tree seedlings has been highly favoured among restoration projects while other growth forms have commonly been disregarded (Ruiz-Jaen & Aide, 2005). Little attention has been paid to non-arborescent plant assemblage development in restoration areas (Garcia et al., 2014; Garcia et al., 2015) as these plants rarely reach desirable diversity in restoration forests in a relatively short time (Shoo et al., 2016; Garcia et al., 2016). Yet these plants can make up a significant portion of biodiversity and have significant ecological functions in their ecosystems.

Epiphytic plants constitute an extremely species-rich guild, including over 27,000 recorded species which amount for almost 10% of global vascular plant diversity (Zotz, 2013), and comprise an essential part of the tropical and subtropical flora (Kreft et al., 2004; Krömer et al., 2005). Lianas also constitute a conspicuous feature in tropical forests, contributing up to 27.1% of their species diversity (Gentry, 1992; Zhu, 2008). Altogether, epiphytes and lianas provide important ecosystem functions, including primary productivity (Clark, Nadkarni & Gholz, 1998), food and habitat provisioning (Duellman, 1988; Nadkarni & Matelson, 1989; Yanoviak, 2015) as well as micro-habitat buffering (Scheffers et al., 2013) and canopy water storage (Campbell et al., 2015; Ah-Peng et al., 2017) among others. However, epiphytes remain understudied (Zotz, 2016; Krömer & Batke, 2025; Sousa da Silva, Freitag Kramer & Zwiener, 2025), especially in secondary forests (Ceballos, 2020), and lianas have also been neglected in many conservation and research programs (Ashton et al., 2001; Nakamura, Proctor & Catterall, 2003; Vargas, Grombone-Guaratini & Morellato, 2020; Stone et al., 2020) despite their importance for ecosystem functioning (Schnitzer, 2015; Gotsch, Nadkarni & Amici, 2016) and their threatened status. Indeed, up to 1,700 liana species could be endangered worldwide (Song et al., 2022) and concerning epiphytes, with the Neotropics as example, 6,721 species (∼60%) are threatened (Carmona-Higuita et al., 2024).

We studied native epiphyte and liana communities growing in tropical forest areas that are currently undergoing ecological restoration for biodiversity conservation after the control of invasive alien plants (Baider & Florens, 2011) on the tropical volcanic oceanic island of Mauritius whose forests are known to sustain advanced invasion (Florens et al., 2016) that often leaves fairly large gaps after the control of invasive plants (Florens & Baider, 2013). In particular, we assessed the epiphyte and liana communities growing on a widespread native pioneer tree species (*Harungana madagascariensis* Lam. (Hypericaceae)) (Bojer, 1837; Baker, 1877; Robson & Stevens, 1980; Botanic Gardens Conservation International (BGCI) & IUCN SSC Global Tree Specialist Group, 2019) and other neighbouring native trees. We selected *H. madagascariensis* as focal species because this tree grows best following the control of woody invasive alien plants like the Strawberry guava (*Psidium cattleyanum*), and is the main native tree species that grow in early stages of secondary ecological succession. However, it is commonly controlled by conservation managers who then typically plant other native species in its place (Florens & Baider, 2013, C. Baider, F.B. Vincent Florens, pers. obs., 2003–2025). More specifically, we compared the potential of *H. madagascariensis* to act as a phorophyte for native epiphytes and lianas relative to other native tree species of (1) comparable diameter, and (2) comparable age to the *H. madagascariensis* to gauge the wider effects of the policy of cutting back the native pioneer tree species from areas undergoing ecological restoration for biodiversity conservation. We investigated three questions in particular to explore the importance of *H. madagascariensis* for native epiphyte and liana communities: (a) How does *H. madagascariensis* compare as a phorophyte with other naturally growing native trees of comparable sizes that belong to species that are commonly planted where *H. madagascariensis* is cut? (b) How does the phorophytic function of *H. madagascariensis* compare with that of other naturally growing trees of comparable age belonging to species that are typically planted after *H. madagascariensis* is removed? (c) Do proxies of fitness of epiphytes and lianas vary depending on whether they grow on *H. madagascariensis* or other phorophytes of comparable ages and of comparable sizes?

## Materials & Methods

### Study sites

Mauritius is a tropical volcanic island located around 900 km east of Madagascar. Centred on 20°15′S and 57°35′E, it is part of the Mascarene Archipelago and falls within the Madagascar and Indian Ocean Islands biodiversity hotspot (Myers et al., 2000). The island is about 1,865 km^2^, with a maximum elevation of 828 m. Mean annual rainfall ranges from 800 mm along the leeward coast to 4,000 mm in the central uplands, with an average annual temperature of 22 °C (Staub, Stevens & Waylen, 2014). Since human colonisation in 1638, extensive habitat transformation has reduced the island’s native vegetation to ∼82 km^2^ or 4.4% of its original extent (Hammond et al., 2015), which nevertheless continues to support a high proportion of native species (Florens et al., 2012). This native vegetation, however, survives as highly fragmented patches (Florens, 2013) that are increasingly dominated by alien woody species, particularly in the understorey (Florens et al., 2016). Although started in the first half of the 20th century (Vaughan & Wiehe, 1941; Baider & Florens, 2025), attempts to restore native vegetation communities by controlling invasive alien plants were upscaled from around the mid-1980s (Strahm, 1993; Jones, 2008), and as of 2024, ∼700 ha of native vegetation (∼8.5% of all native remnants) is undergoing restoration in that way on the island (Republic of Mauritius, 2024).

**Figure 1:**
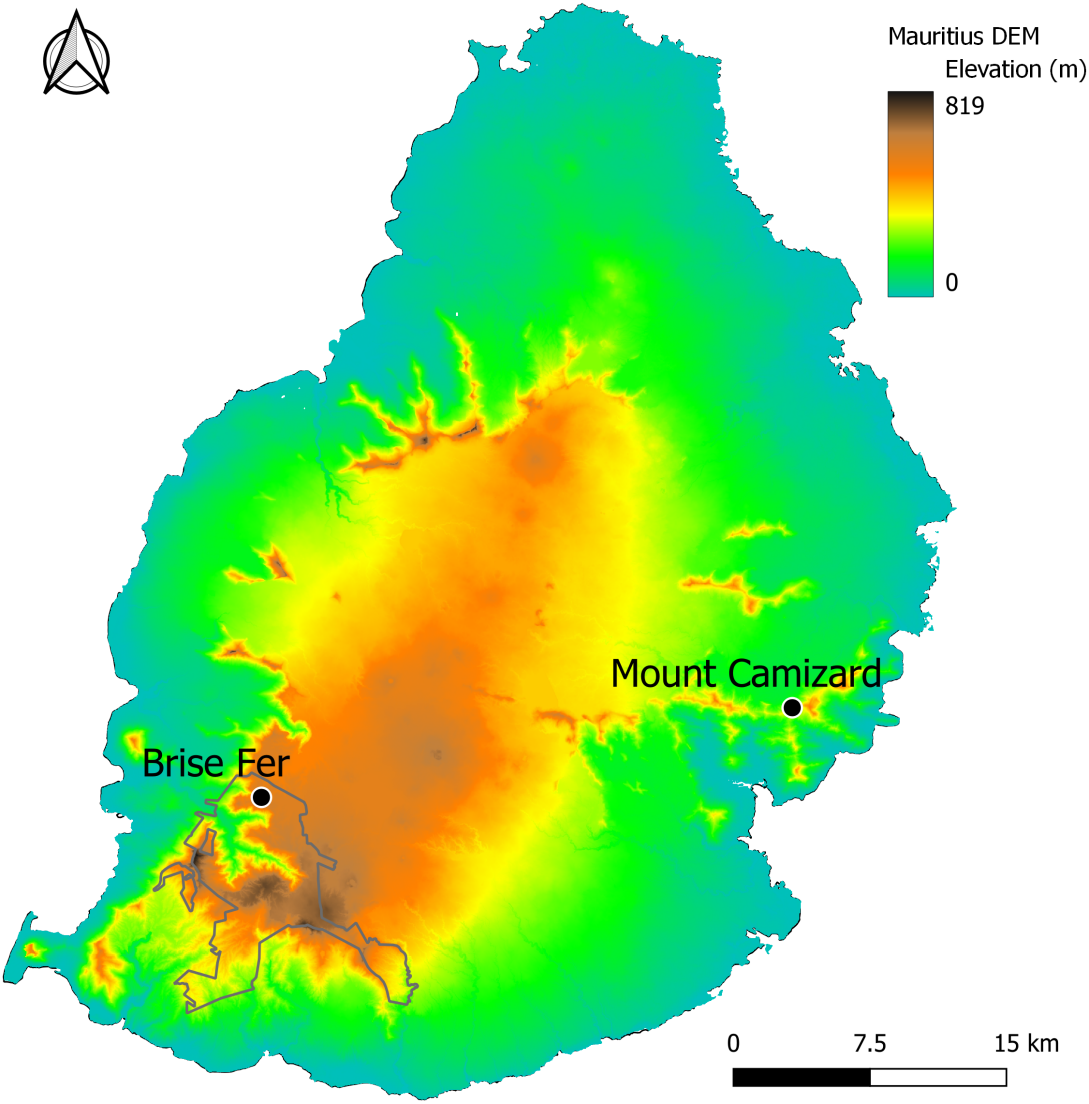
The study sites of Brise Fer and Mount Camizard in Mauritius displayed on a digital elevation model (DEM) showing topographic variation. The Black River Gorges National Park is outlined. Mount Camizard is found within protected Mountain Reserves.

Two of the forest areas undergoing ecological restoration for biodiversity conservation and known to host native epiphyte and liana communities (Fig. 1) were surveyed between August 2023 and August 2024. The first one, Mount Camizard, (20°19′51″to 20°20′00″S and 57°42′52″to 57°43′02″E, 250–320 m asl) is located in the island’s South-East within an area of Mountain Reserve inside the Bamboo Mountains forest block. The native forest at the site has been undergoing ecological restoration mainly through invasive alien plant control since 2005 and is close to the lower elevational range of *H. madagascariensis* on Mauritius. The second site, Brise-Fer, occurs close to the higher elevational range of *H. madagascariensis* in the South-West of Mauritius (20°22′10″to 20°22′30″S and 57°25′55″to 57°26′20″E, 560–600 m asl) within the Black River Gorges National Park. There, weeding of invasive alien plants has been done in different phases for conservation management since 1986. We selected the largest area, weeded in 1996, for our sampling. Mount Camizard and Brise Fer receive comparable annual rainfall of respectively 2.5 and 2.4 m, and no permanent water sources, but some storm streams, cross the study areas. Surveys in the Black River Gorges National Park and Mount Camizard were conducted with permission from The National Parks and Conservation Services of the Ministry of Agro-Industry, Food Security, Blue Economy and Fisheries, and Mr. Owen L. Griffiths and Mrs. Mary-Ann Griffiths respectively.

### Data collection

**Figure 2:**
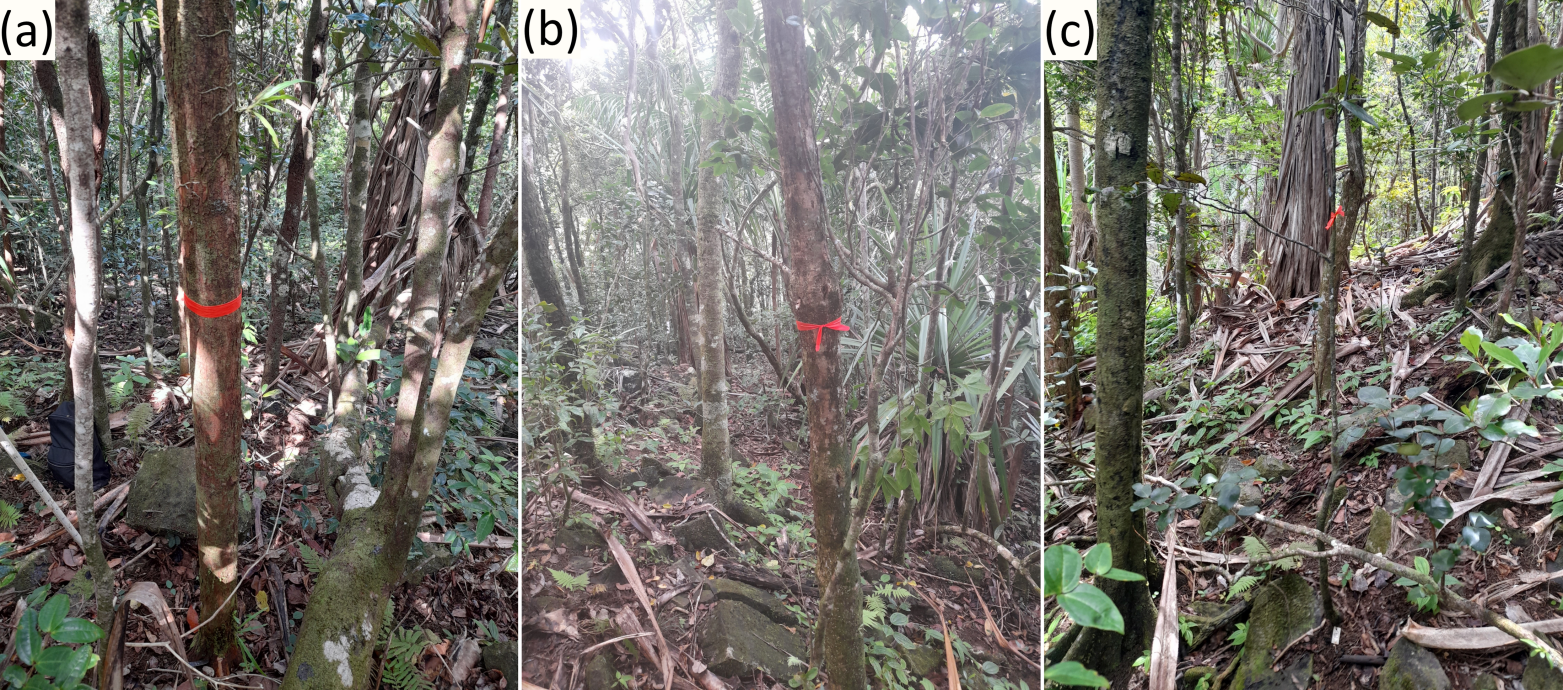
Illustration of phorophytes sampled in Mount Camizard. (A) One *Harungana madagascariensis* of 9.5 cm, with (B) the closest individual (2.3 m distance) of another native species (*Euphorbia pyrifolia*) of similar diameter (9.5 cm), and (C) the closest individual (here at 1 m distance) of another native species (*Diospyros tessellaria*) of similar age (trunk diameter = 0.97 cm) (section ‘Estimation of tree age’ explains how similar ages were determined). Photos: François Baguette.

**Figure 3:**
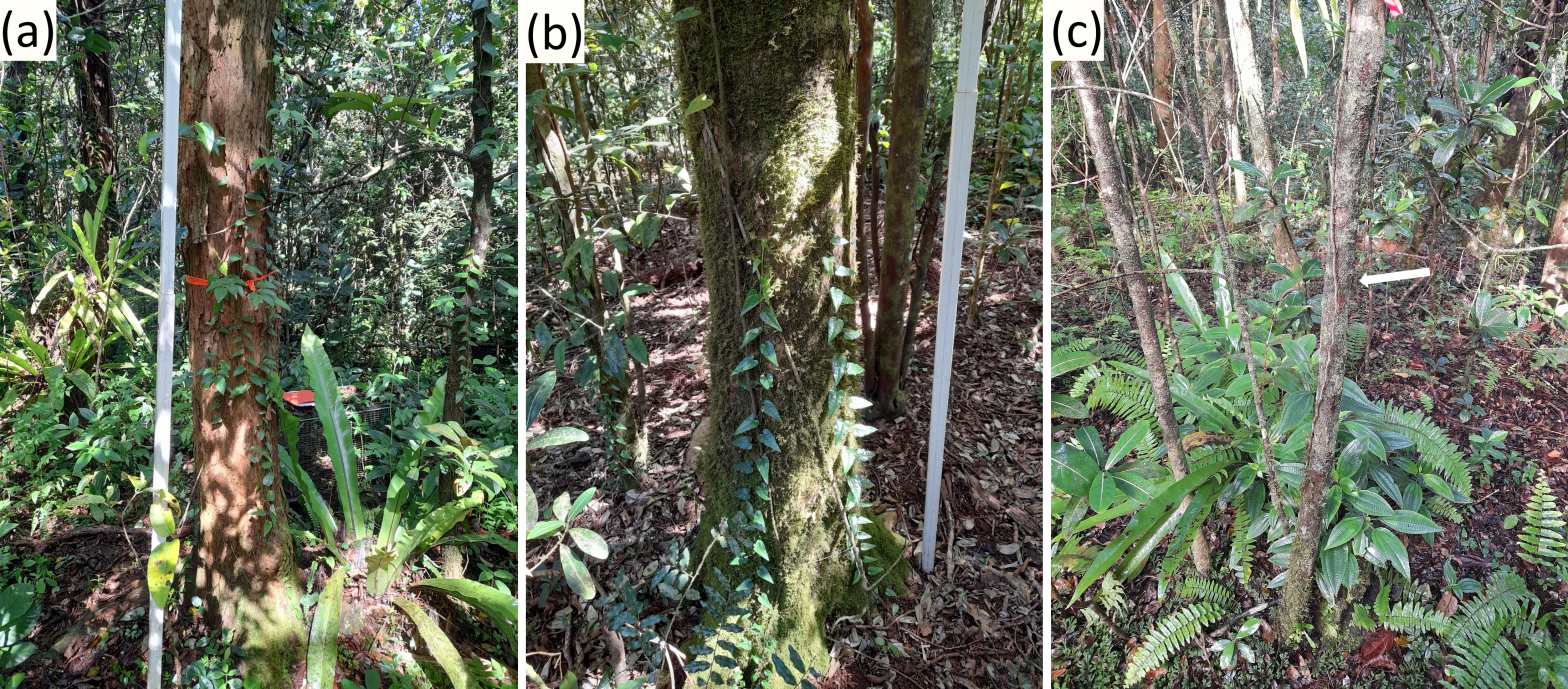
Illustration of phorophytes sampled in Brise Fer. (A) One large *Harungana madagascariensis* of 30 cm trunk diameter, with (B) the closest individual (12 m away) of another native species (*Psiloxylon mauritianum*) of similar trunk diameter (31.9 cm), and (C) the closest individual (indicated by arrow) of another native species (*Eugenia kanakana*) of similar age (trunk diameter = 4.5 cm) (section ‘Estimation of tree age’ explains how similar ages were determined). Photos: François Baguette.

Naturally growing (non-planted) trees of *H. madagascariensis* (52 at Mount Camizard and 21 at Brise Fer) were randomly sampled along with the closest individual of another native tree species to each of them that was of (1) similar trunk diameter (measured at 1.3 m above ground, along the stem) and (2) similar age (Figs. 2 and 3). The decision to focus on individual trees rather than specific host species was in part guided by previous studies demonstrating that epiphyte assemblages are often more strongly influenced by tree-level structural attributes than by host identity (Zotz & Vollrath, 2003; Flores-Palacios & García-Franco, 2006; Komada et al., 2022), and that host specificity among vascular epiphytes and lianas is generally weak or absent (Garrido-Perez & Burnham, 2010; Wagner, Mendieta-Leiva & Zotz, 2015). The choice to include tree age in our analysis was guided by previous studies demonstrating the importance of including the dynamic of habitat-building trees in epiphyte studies (Merwin, Rentmeester & Nadkarni, 2003; Wagner & Zotz, 2020) and to ensure the validity of our comparisons between trees of different ecology and longevity. In Mount Camizard, each potential *H. madagascariensis* tree was selected using a coin-flip method, whereas at Brise Fer, trees were chosen from individuals previously recorded in a long-term monitoring plot of one hectare using random number attribution.

Trees of similar age were chosen from within the ten most important species of each site (Florens et al., 2012) to ensure their nearby occurrence, their most comparable ecological importance to *H. madagascariensis* which itself is relatively common, and to also reflect the most commonly planted species by conservation practitioners after they cut *H. madagascariensis*. In all, 219 woody plants were sampled, including 73 trees of each category, which served as the sampling units. For each of the 219 potential phorophytes, GPS coordinates were recorded (with GPSMap^®^ 65, Garmin), the species identified, and its diameter at breast height (DBH) recorded. Plant reproductive status and size were used as proxies of epiphytes and lianas’ fitness following Tremblay et al. (1998), Zhang, Nurvianto & Harrison (2010) and Wagner & Zotz (2020). The reproductive status of each epiphyte and liana was recorded as either vegetative or reproductive (bearing flower buds, open flower, unripe fruit, ripe fruit or showing traces of fallen fruits for angiosperms and fertile fronds for pteridophytes). The number of leaves or fronds of each epiphyte, and the stem diameter of each liana, were recorded to assess plant size.

Vascular epiphytes and lianas were counted on all sampled trees from the ground, with the aid of an 8 × 42 pair of binoculars when necessary, and identified using identification keys from the regional Flora (Bosser et al., 1976–2023) and other relevant work (Hermans et al., 2025) (illustrations of selected species are provided in supplementary material (Fig. S1)). Identifications were validated by the second author, CB, and third author, FBVF, who are experts in Mauritius plant taxonomy. Sampled trees ranged from 1.3 m to 15.0 m in height, with *H. madagascariensis* reaching on average similar heights than other native trees of similar diameter (8.98 m and 8.36 m respectively). Trees of similar age were on average 3 m high. For larger trees (>8 m height), we restricted our census up to the first section of the canopy (*e.g.*, 1/3 of the branches length), equivalent to “Zone 3” (Johansson, 1974), to avoid observation bias due to the high probability of missing individuals higher up. Hemiepiphytes and hemiparasites were not considered due to their different ecology from epiphytes and lianas and also because of their rarity or absence in the study areas. We defined an individual epiphyte as an assemblage of rhizomes and leaves forming a clearly bounded stand (Sanford, 1968) due to the difficulty of delineating individual epiphytes when multiple shoots occur in close proximity. For species exhibiting a creeping growth form, individuals were considered separate if physically separated rhizome segments were growing on distinct branches or if no visible connection was discernible between them. Finally, each clearly separated clump of epiphytic filmy ferns (Hymenophyllaceae) observed was defined as a single individual due to the impossibility to delimitate individuals otherwise in the field.

### Estimation of tree age

We estimated tree age using the best long-term monitoring census data available for the island supplied by the Mauritius Herbarium and comprising ∼19,000 individual native woody plants belonging to ∼100 species from Brise Fer. It includes tree measurements from 2005, 2010 and 2022 (spanning a maximum of 17 years) collected in 3 ha permanent plots, which provided the most reliable results compared to the smaller datasets available (including from Mount Camizard) that were collected during a shorter time period and that would therefore include a larger margin of error. Furthermore, the similarity in climate and forest structure and composition between the two sites and the extremely low growth rate of the studied species (0.15 cm/year on average, see Table S1) limits the potential bias that the lack of long-term data from Mount Camizard could have on our results. We calculated the annual DBH increments of *H. madagascariensis* and of the ten most important species along which it grows in both study sites using two segments of stem diameter monitoring (from 2005–2010 and from 2010–2023). The ten most important species were determined following Importance Values from Florens et al. (2012). Changes in stem diameters were used to estimate the average annual growth rate of each species of interest. A growth rate ratio between *H. madagascariensis* and each of the other species was then computed to estimate the diameter of an individual of the ten other most important species that would be of similar age to the individual of *H. madagascariensis* being sampled (Table S1). Finally, we sampled the nearest similar-aged plant to each *H. madagascariensis* studied. This indirect method was chosen as the census data used were the best available data for tree age estimation, and more reliable methods such as tree coring could not be performed due to the risk of damage (Florens, 2014; Tsen, Sitzia & Webber, 2016) which would not have been justifiable in our context.

### Data analysis

Only native species were used in the data analysis, because introduced species encountered represented only individuals that were missed during invasive alien plant control campaigns and are therefore not characteristic of the study sites, and transient in nature until removed at a future weeding campaign. In all, 81% (1,805 of 2,229 individuals) of all epiphytes and lianas observed on the 219 sampled potential phorophytes were identified to species level directly *in situ* or at the National Herbarium of Mauritius based on photographs taken *in-situ*. Beside these, Hymenophyllaceae were treated as a single group due to their small size and the difficulty to identify them to species level. Observations were grouped under morphospecies groups for species that were indistinguishable from each other either because of the lack of distinctive characters on immature individuals or due to the absence of visible distinctive characters. This was the case for *Angraecum calceolus*, *Angraecum caulescens* and *Angraecum multiflorum* which have been grouped under the “*Angraecum* spp. Group*”* (*n* = 383); *Bulbophyllum* spp. grouped under the “*Bulbophyllum* spp. Group*”* (*n* = 4); *Haplopteris* spp. in “*Haplopteris* spp. Group*”* (*n* = 3), *Polystachia mauritiana* and *Polystachia virescens* grouped in “*Polystachia mauritiana s.l.* Group” (*n* = 119), and *Selaginella* spp. in “*Selaginella* spp. Group*”* (*n* = 19). Four observations of ferns could not be associated to any genus and those were excluded from the data analysis, on the basis that they could have been immature alien species. The final dataset used for analysis comprised the respective taxa classified as morphospecies, with 2,229 records of epiphytes and lianas collected from 219 sampled phorophytes.

We used R version 4.4.1 (R Core Team, 2024) to do all statistical analysis and graphs. Hill numbers were calculated per tree across phorophyte category and site (orders *q* = 0, 1, 2) using the R package ‘HillR’ (Li, 2018) to capture species richness, effective number of common species (Shannon), and dominant species (Simpson) (Hill, 1973; Chao, Chiu & Jost, 2014). Structural variables such as epiphyte abundance (N, epiphytes tree^−1^), and species richness were also computed. We assessed differences in epiphyte species richness and abundance among tree categories (defined by diameter at breast height) using generalized linear models (GLMs), with tree category as the main predictor, and site as a covariate. Counts of epiphytes were explored for overdispersion and excess zeros using residual diagnostics using ‘DHARMa’ package (Hartig, 2024). Species richness was analysed using Quasi-Poisson GLMs, while abundance was modelled using zero-inflated negative binomial (ZINB) GLMs to account for overdispersion and excess zeros. Model fit was evaluated using pseudo *R^2^* (1 − residual deviance/null deviance) (Zuur et al., 2009). *Post hoc* pairwise comparisons between tree categories were performed using estimated marginal means (EMMs) with Tukey adjustment *via* the ‘emmeans’ package (Lenth, 2025). To evaluate differences in phorophyte suitability for epiphytes, total abundance and total species richness of all epiphyte and liana individuals per tree were used as response variables. Graphs were produced using the R package ‘ggplot2’ (Wickham, 2016).

Description of the epiphyte and liana communities in each tree category was made through its species composition and the relative importance value of all species. To this end, we carried out an indicator species analysis using the multipatt function in the indicspecies package (version 1.7.15) (De Cáceres, Legendre & Moretti, 2010) to identify species that are good indicators for one or several tree categories (Dufrêne & Legendre, 1997). In addition, we chose the Orchidaceae, the most abundant epiphyte family recorded (1,181 individuals), to compare the abundance of reproductive individuals (N, reproductive orchids tree^−1^) growing on *H. madagascariensis* and other phorophytes of similar age and size. The most abundant orchid species, namely *Angraecum pectinatum* (523 individuals), and the most abundant species of liana recorded, namely *Piper borbonense* (226 individuals) were used to compare the sizes of epiphytes and lianas growing on the different phorophytes. Extreme values of reproducing orchid abundance and plant size were removed prior to analysis to reduce the influence of extreme outliers. The distribution of reproductive status and plant size data was assessed using the Shapiro–Wilk test. As the data deviated from normality, non-parametric tests were applied in subsequent analyses. Reproductive orchid abundance and the size of epiphyte/liana were compared between the different tree categories using Kruskal–Wallis rank sum tests with the *post-hoc* Dunn’s tests of multiple comparisons with Bonferroni adjustment using the R packages ‘rcompanion’ and ‘dunn.test’ (Mangiafico, 2024; Dinno, 2024). Graphs were produced using the R package ‘ggplot2’ (Wickham, 2016).

## Results

Epiphytes and lianas were recorded on 116 of 219 (53%) sampled potential phorophytes, including very small ones (DBH ∼1cm). Trees devoid of epiphytes or lianas were mostly of relatively small sizes (median DBH: 5.5 cm), but also included six relatively large trees (DBH ≥ 15 cm). Plants sampled hosted 23 epiphyte species (1,973 individuals) and five liana species (256 individuals) (Table S2). Overall, half (14) of the species occurred as <10 individuals each, and the other half was represented by >2,000 individuals (Table S3). The most abundant species was the orchid *Angraecum pectinatum*, which accounted for 23.5% of all epiphytes. Other *Angraecum* species grouped into ‘*Angraecum* spp.’ and the fern *Microsorum punctatum* were the other most frequent epiphytes (Table S4). The Orchidaceae was the most important plant family, both in terms of abundance and species richness, including 36% of all species or taxa and 53% of all individuals (Table S5).

**Figure 4:**
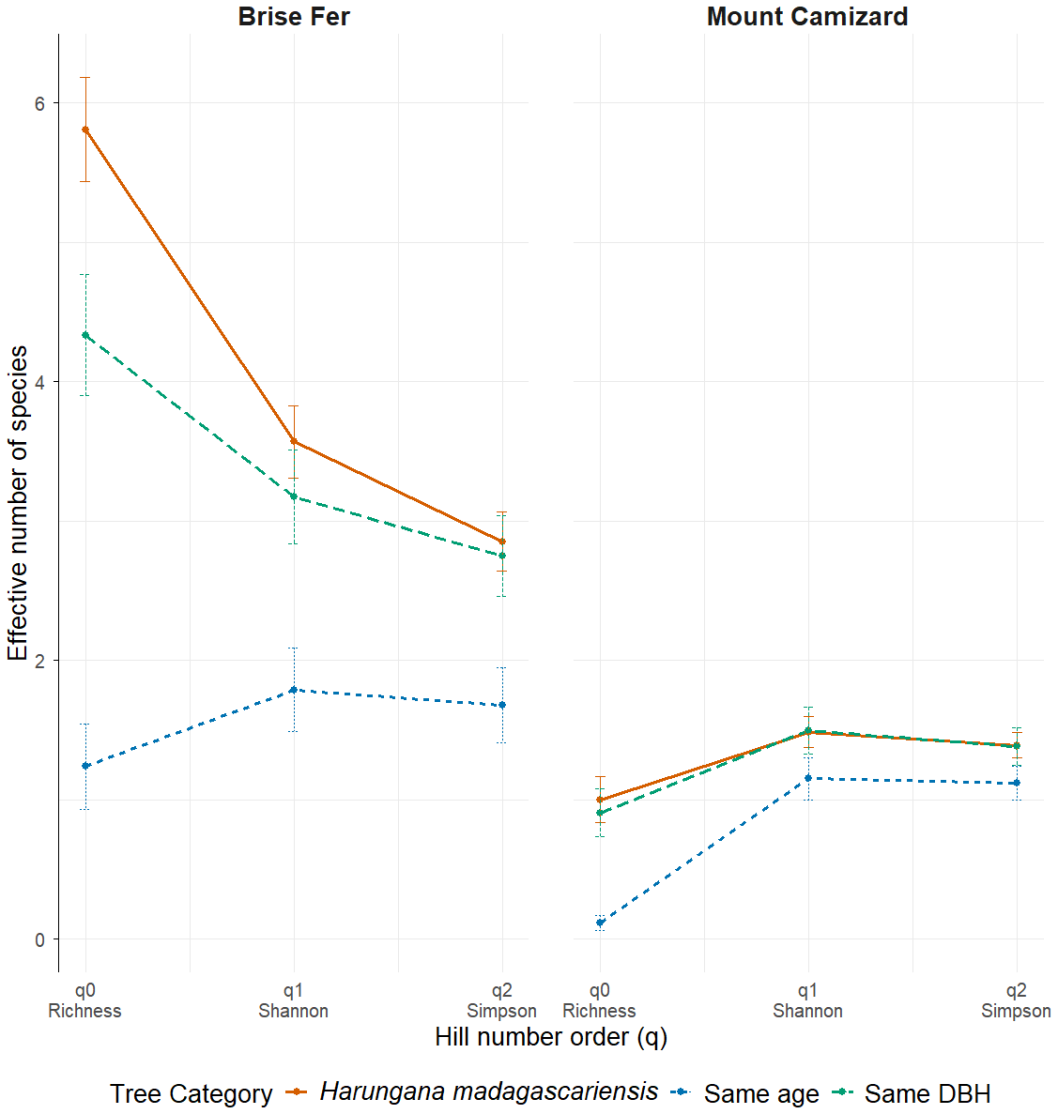
Effective number of species (Hill numbers) per tree across phorophyte category and site. q0 represents the mean species richness, q1 the mean effective number of equally common species, and q2 the mean effective number of dominant species. ‘Same DBH’ stands for other potential native phorophytes having comparable diameter at breast height and paired with each *H. madagascariensis* sampled; and ‘Same age’ refers to other potential native phorophytes having comparable age and paired with each *H. madagascariensis* sampled.

**Figure 5:**
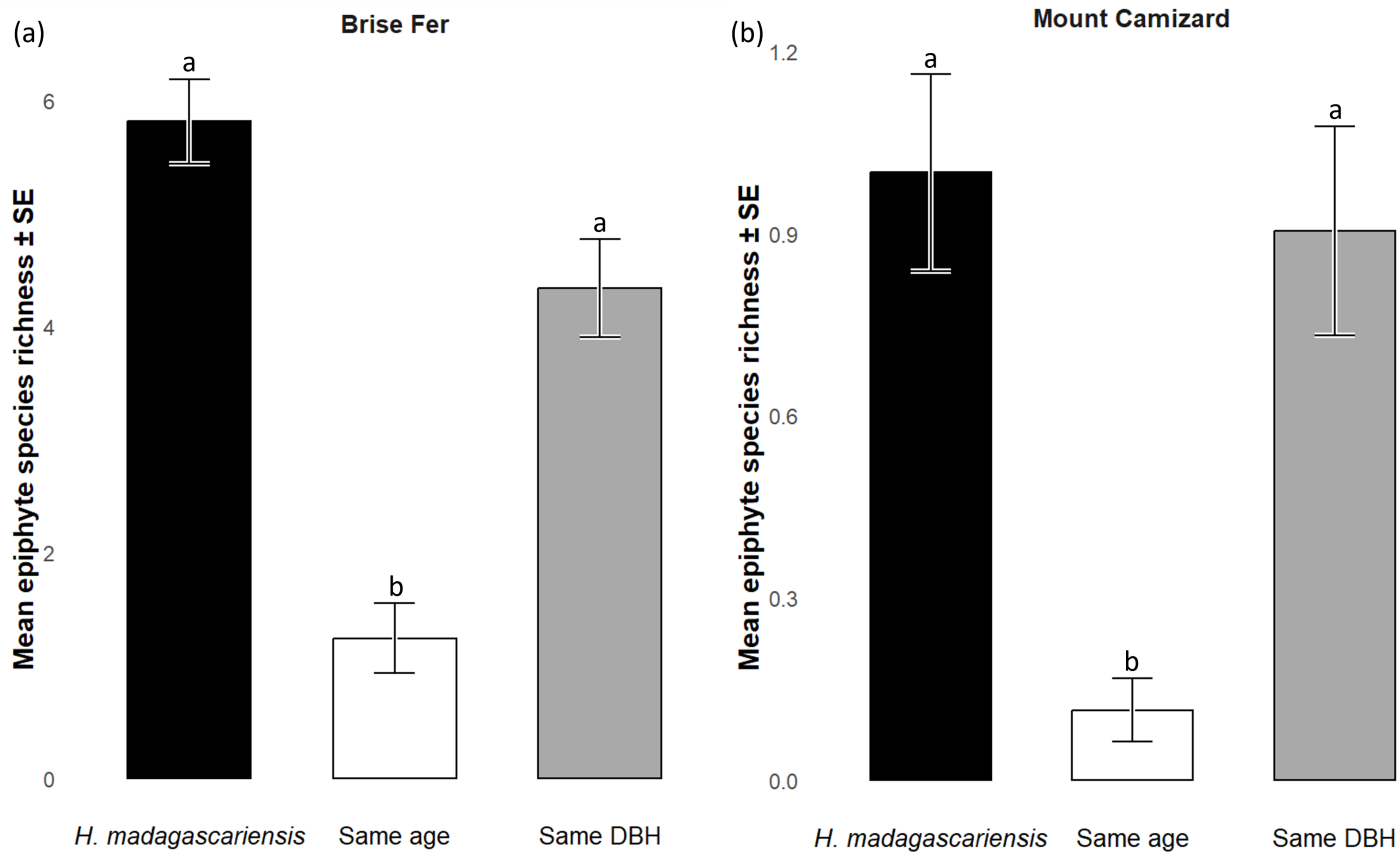
Species richness (± SE) on *Harungana madagascariensis* and other potential phorophytes in (A) Brise Fer and (B) Mount Camizard where native forests are undergoing ecological restoration after weeding of invasive alien plants. ‘Same DBH’ stands for other potential native phorophytes having comparable diameter at breast height and paired with each *H. madagascariensis* sampled; and ‘Same age’ refers to other potential native phorophytes having comparable age and paired with each *H. madagascariensis* sampled. “Epiphyte” refers to both epiphyte and liana species. Different letters above bars indicate significant differences among categories, whereas bars sharing the same letter are not significantly different.

**Figure 6:**
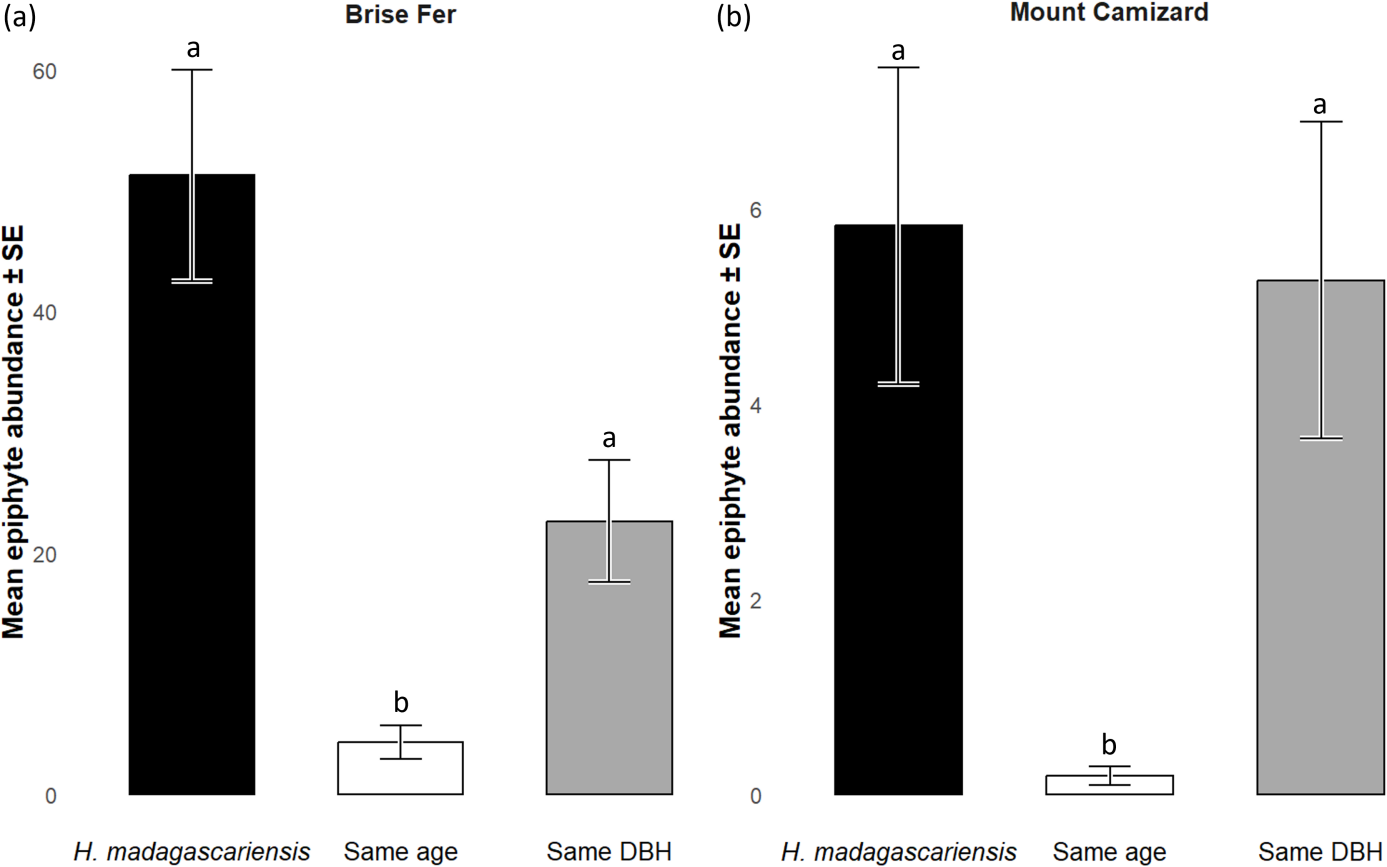
Abundances of native epiphytes (± SE) on *Harungana madagascariensis* and other phorophytes in (A) Brise Fer and (B) Mount Camizard where native forests are under ecological restoration after weeding of invasive alien plants. ‘Same DBH’ stands for other potential native phorophytes having comparable diameter at breast height and paired with each *H. madagascariensis* sampled; and ‘Same age’ refers to other potential native phorophytes having comparable age and paired with each *H. madagascariensis* sampled. “Epiphyte” refers to both epiphyte and liana species. Different letters above bars indicate significant differences among categories, whereas bars sharing the same letter are not significantly different.

Generally, *H. madagascariensis* hosted a higher diversity of epiphyte and liana than other native trees of (1) similar age and (2) similar size, a situation more pronounced in Brise Fer than in Mount Camizard (Fig. 4). Tree category significantly affected epiphyte species richness (*χ*^2^ = 90.403, *df* = 2, *p* < 0.05), as well as site (*χ*^2^ = 192.156, *df* = 1, *p* < 0.05). *Post-hoc* estimated marginal means (emmeans) indicated that *H. madagascariensis* hosted significantly more epiphyte species than trees of similar age (*p* < 0.05), but differences with trees of similar diameter were not significant (*p* > 0.05) (Fig. 5). Epiphyte abundance was also significantly affected by tree categories (*χ*^2^ = 92.243, *df* = 2, *p* < 0.05), as well as site (*χ*^2^ = 65.177, *df* = 1, *p* < 0.05). *Harungana madagascariensis* hosted significantly more epiphytes than trees of similar age (*p* < 0.05) but differences with trees of similar diameter were not significant (*p* < 0.05) (Fig. 6).

**Figure 7:**
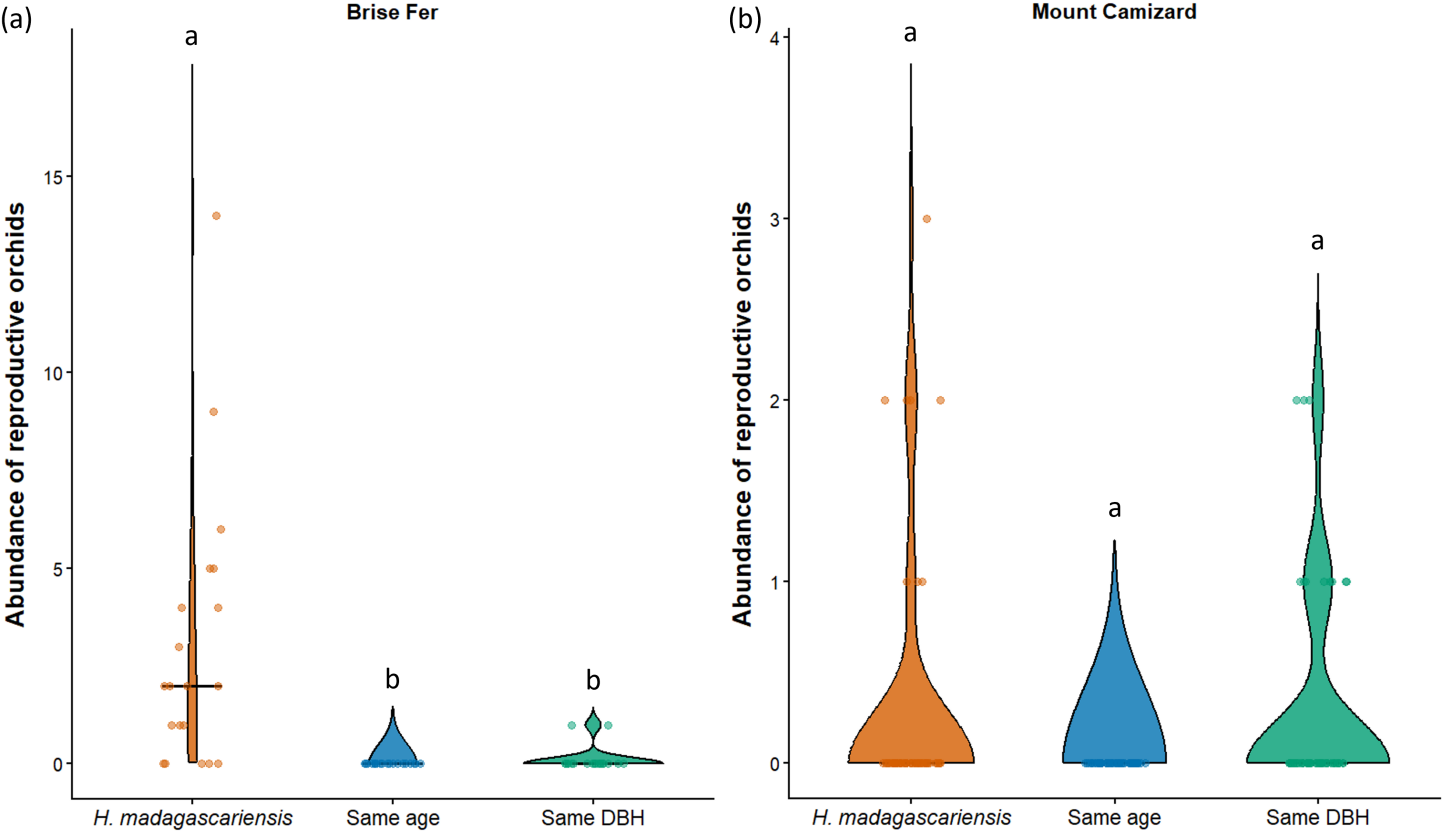
Abundance of reproductive orchids on *Harungana madagascariensis* and other phorophytes at the two sampled sites: (A) Brise Fer and (B) Mount Camizard, where native forests are undergoing ecological restoration. Violin plots show the distribution density of orchid abundance per phorophyte category. The central horizontal lines indicate the medians, and individual observations are shown as jittered points. ’Same DBH’ stands for other potential native phorophytes having comparable diameter at breast height and paired with each *H. madagascariensis* sampled; and ’Same age’ refers to other potential native phorophytes having comparable age and paired with each *H. madagascariensis* sampled. Different letters above bars indicate significant differences among categories, whereas bars sharing the same letter are not significantly different.

In Brise Fer, six out of 27 sampled species were significantly associated with *H. madagascariensis* compared to only three with other trees of similar diameter. Furthermore, five species were significantly associated with *H. madagascariensis* and trees of similar diameter compared to only one being associated with the combination of *H. madagascariensis*, trees of similar diameter, and trees of similar age (Table 1). No significant association were found in Mount Camizard. In terms of fitness, there was a significant difference in abundance of reproductive orchid (*χ*^2^ = 70.52, *df* = 5, *p* < 0.05) among phorophytes across both sites, with *post hoc* Dunn’s tests revealing that *H. madagascariensis* hosted significantly more reproductive orchid than other native phorophyte of (1) similar age and (2) similar size in Brise Fer (*p* < 0.05 respectively) but no significant difference was observed in Mount Camizard (Fig. 7). There was no significant difference of size (in terms of number of leaves) for *Angraecum pectinatum* (*χ*^2^ = 4.43, *df* = 4, *p* > 0.05) among phorophytes across both sites. With regards to lianas, there was no significant difference of size (in terms of stem DBH) for *Piper borbonense* among phorophytes in Brise Fer (*χ*^2^ = 1.11, *df* = 2, *p* > 0.05).

**Table 1:** Native epiphytes and lianas significantly associated with potential phorophytes in Brise Fer where native forest is undergoing ecological restoration after weeding of invasive alien plants. ‘*Harungana*’ stands for *Harungana madagascariensis*; ‘Same DBH’ stands for other potential native phorophytes having comparable diameter at breast height and paired with each *H. madagascariensis* sampled; and ‘Same age’ refers to other potential native phorophytes having comparable age and paired with each *H. madagascariensis* sampled.

Species	Site	Associated phorophyte(s)	IndVal	*p*value
*Angraecum pectinatum*	Brise Fer	*Harungana*	0.919	<0.05
*Cnestis polyphylla*	Brise Fer	*Harungana*	0.612	<0.05
*Nephrolepis cordifolia*	Brise Fer	*Harungana*	0.593	<0.05
*Angraecum mauritianum*	Brise Fer	*Harungana*	0.504	<0.05
*Polystachia mauritiana s.l.*	Brise Fer	*Harungana*	0.430	<0.05
*Bulbophyllum* spp.	Brise Fer	*Harungana*	0.309	<0.05
*Asplenium nidus* var. *nidus*	Brise Fer	Same DBH	0.412	<0.05
*Urera acuminata*	Brise Fer	Same DBH	0.404	<0.05
Hymenophyllaceae	Brise Fer	Same DBH	0.378	<0.05
*Piper borbonense*	Brise Fer	*Harungana* + Same DBH	0.748	<0.05
*Microsorum punctatum*	Brise Fer	*Harungana* + Same DBH	0.614	<0.05
*Selaginella* spp.	Brise Fer	*Harungana* + Same DBH	0.488	<0.05
*Nephrolepis biserrata*	Brise Fer	*Harungana* + Same DBH	0.408	<0.05
*Rumohra adiantiformis*	Brise Fer	*Harungana* + Same DBH	0.345	<0.05
*Lepisorus spicata*	Brise Fer	*Harungana* + Same DBH + Same age	0.647	<0.05

## Discussion

### Ecological implications

The native pioneer tree *H. madagascariensis* which grows naturally best in disturbed areas, precisely where other potential phorophytes are rare, constitutes a better phorophyte compared to other potential native phorophytes that grow alongside it in the Mauritian native forests. Furthermore, the epiphyte and liana communities that *H. madagascariensis* can support in just two to three decades of its growth, are comparable to those assembling on often much-slower growing and much older, often multi-centennial trees of comparable size to *H. madagascariensis*. This further stresses the importance of *H. madagascariensis* for epiphytes and lianas establishment following a disturbance, reinforcing previous findings showing that tree age and size strongly influence phorophytic function in various ways that depend on tree species’ ecology (pioneer *versus* later successional species) (Catling & Lefkovitch, 1989; Wolf, 1994; Annaselvam & Parthasarathy, 2001; Bernal, Valverde & Hernández-Rosas, 2005; José Válka Alves, Kolbek & Becker, 2008).

Moreover, we showed that, within two to three decades of a disturbance, the fitness of native epiphytes (as assessed using commonly used proxies of fitness) can be substantially superior for those establishing on *H. madagascariensis* compared to other potential phorophytes close by. This double superiority as phorophyte (hosting greater diversity of epiphytes and fostering their higher fitness) is apparent even when compared to much older other species of the same trunk diameter as the *H. madagascariensis*. This situation appears linked to the fact that the bark of *H. madagascariensis* is relatively thick and spongy compared to most other native phorophytes. Such a bark retains moisture for longer periods and probably provide more nutrients, thereby promoting epiphyte establishment and their faster growth and maturation compared to most other Mauritian native trees. Hence, *H. madagascariensis* can not only quickly provide large surface areas suitable for epiphyte establishment and maintenance, but also offer a suitable habitat for their relatively rapid growth and earlier maturation. Those results corroborate previous studies showing that pioneer trees can be suitable phorophytes for epiphytes (Callaway et al., 2002; Cascante Marín et al., 2008; Einzmann et al., 2015; Besi et al., 2023; Pie et al., 2023; Wysocki et al., 2024), and lianas (Putz, 1984; Letcher, 2015; Schnitzer, 2015). However, it is important to note that site conditions modulate the benefits that pioneer trees like *H. madagascariensis* can bring, with greater positive impacts on boosting epiphytes and lianas in sites where greater species richness and abundance of epiphytes and lianas are found (*e.g.*, Brise Fer compared to Mount Camizard).

Finally, the indicator analysis identified 21.4% of the epiphyte and liana species recorded in this study (*N* = 6) as significantly associated with *H. madagascariensis*, compared with only 10.7% (*N* = 3) with trees of the same size. These proportions are consistent with previous studies reporting that only a minority (15–30%) of epiphyte species show non-random host associations (Laube & Zotz, 2006; Wagner, Mendieta-Leiva & Zotz, 2015; Wagner, Wanek & Zotz, 2021), but further stress the important role that *H. madagascariensis* plays in supporting specific native epiphyte and liana species compared to other native trees that grow in conditions closest to those where *H. madagascariensis* grows. Therefore, *H. madagascariensis* trees provide a suitable habitat for epiphytes and lianas relatively early following a disturbance. Importantly, the patterns observed in Mauritius are likely applicable across the extensive native range of *H. madagascariensis* (over 12 million km^2^; Baguette, Baider & Florens, 2025), wherever the species occurs within the natural distribution of epiphytic orchids, ferns, and lianas, given the broad similarity in the ecological niches and requirements of these plant guilds.

### Implications for ecological restoration and biodiversity conservation

**Figure 8:**
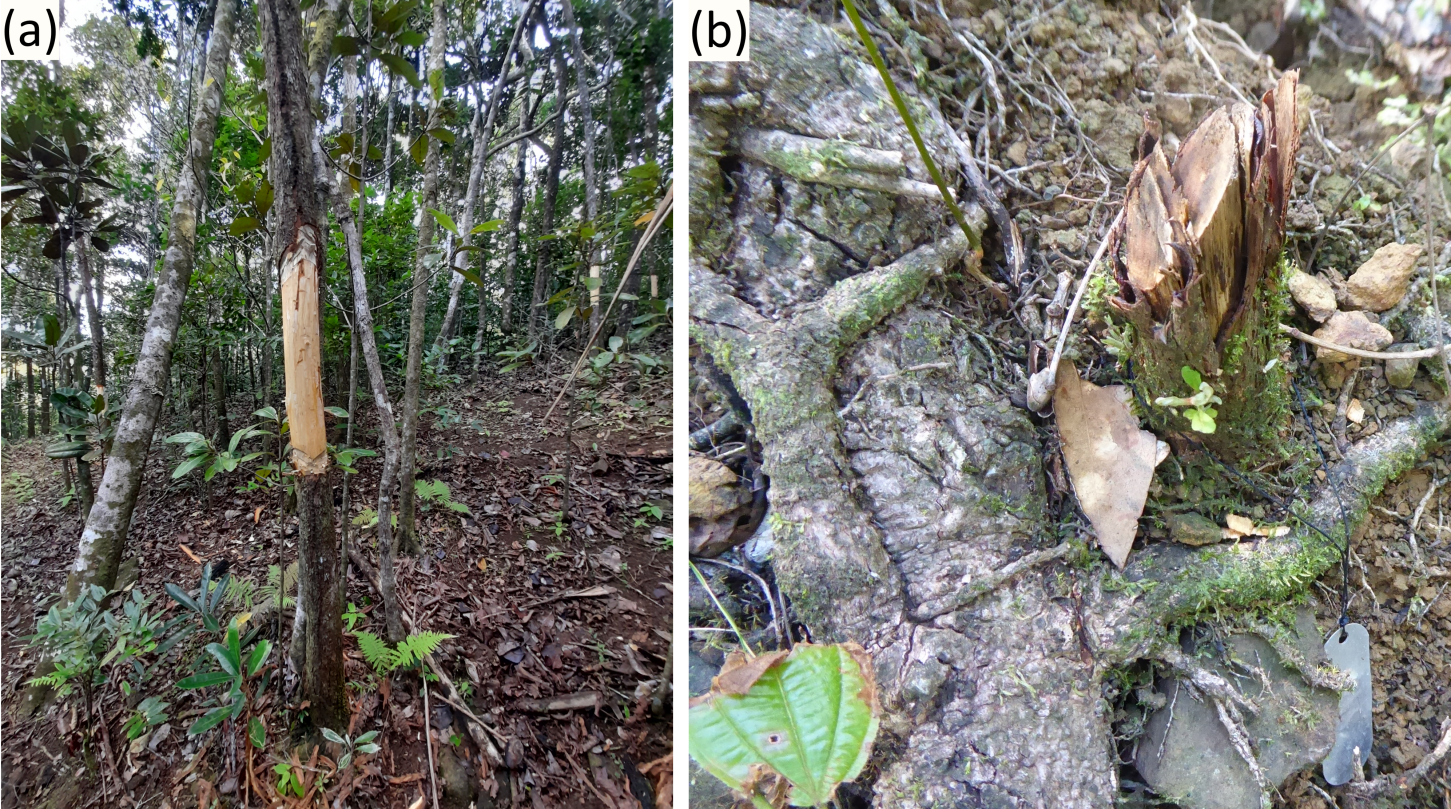
Control of *Harungana madagascariensis* within native forests undergoing restoration for conservation of biodiversity done alongside maintenance weeding of invasive alien plant species. (A) Ring-barked *H. madagascariensis* observed at Mount Camizard during data collection for this study. (B) Cut stem of *H. madagascariensis* (whose growth was being monitored – note metal tag) observed in a different restoration area managed by a different organization located in Ferney in Mauritius. Photos: François Baguette.

The extreme invasion of Mauritius native forests by alien plants (Florens et al., 2016) has driven a high rate of native tree mortality (Florens et al., 2017) including some of the largest canopy species (Baider & Florens, 2006). As a result, when alien plants are removed, scanty native trees often remain to foster ecological restoration within the substantial gaps created in the forest canopy. These gaps form ideal habitat for *H. madagascariensis* which grows naturally from the seedbank to recreate a canopy reaching ∼12 m high within four to six years (Swaine & Hall, 1983; Ndam & Healey, 2001; Manjaribe et al., 2013) before starting to decline after ∼10 years (Hervé et al., 2015). Our results show that, where it grows, this pioneer tree does not suppress native epiphytes and lianas but on the contrary, is highly beneficial to native epiphytes species richness, abundance and fitness, more so than other species that grow alongside it. Yet, all major conservation practitioners of Mauritius have been, at one point or another, cutting back large numbers of *H. madagascariensis* in areas undergoing ecological restoration for biodiversity conservation (Fig. 8), contra best available evidence of its benefits for ecological restoration (Florens & Baider, 2013; Baguette, Baider & Florens, 2025), and without evidence of its presumed negative impact on native biodiversity. Here, we show that in addition to such practices hindering restoration progress of woody plant cover and fostering re-invasion by invasive alien plants (Florens & Baider, 2013), it also slows broader ecosystem recovery by: (1) removing native structural epiphytes already established on *H. madagascariensis*; and (2) reducing future recruitment of structural epiphytes, as alternative phorophytes generally provide lower-quality habitat than *H. madagascariensis*.

Among the species significantly associated with *H. madagascariensis*, four are orchids. Orchids constitute a major group of the island flora as it is the island’s most species-rich family of flowering plants, and is dominated by species endemic to the biodiversity hotspot region (80%), followed by species endemic to the Mascarene archipelago (41%), including those endemic to the island (10%) (Baider & Florens, 2022). The Orchidaceae is also the native angiosperm family that has sustained the highest extinction rate on Mauritius, with 22% of Mauritian native orchids driven extinct over the last 2.5 centuries or so (Baider & Florens, 2022) and many species are now extremely rare (*e.g.*, Baider et al., 2012; Pailler et al., 2020a). Furthermore, known species have been found for the first time on the island relatively recently (Roberts et al., 2004) and new species are still being discovered even more recently (Fournel, Micheneau & Baider, 2015; Pailler & Baider, 2020; Pailler et al., 2020b). For all these reasons, conservation of native orchids in Mauritius should be a priority and our results show that *H. madagascariensis* can greatly help to enhance their conservation in wet forests by providing advantageous habitats for their colonisation and maturation. It is thus particularly unfortunate that most conservation managers cut back *H. madagascariensis* from areas undergoing restoration. Importantly, *H. madagascariensis* germinates and grows naturally in wet forests undergoing restoration such that no additional investment after invasive plants weeding is required from conservation managers for its establishment.

Finally, it is important to stress that epiphyte support diverse ecological interactions with animals (Nadkarni & Matelson, 1989; Stuntz et al., 2002; Boechat, da Silva & Nunes-Freitas, 2019; Spicer & Woods, 2022), as lianas also do (Yanoviak, 2015; Odell, Stork & Kitching, 2019). In particular, *Piper borbonense*, the most abundant native liana growing on *H. madagascariensis*, produces many fleshy fruits eaten by and benefiting native vertebrates (Heinen et al., 2023) including the threatened endemic Mauritius Bulbul (*Hypsipetes olivaceus*), also recently found to be a key pollinator of a fast declining endemic plant (Bissessur et al., 2019), thereby stressing on the judiciousness of considering broader ecological interconnectivity when devising ecological restoration policy. Mauritius is the only place within *H. madagascariensis*’ ∼12 million km^2^ native range where conservationists cut the tree (Baguette, Baider & Florens, 2025), based on justifications contradicting best available evidence, including the unsubstantiated claim that it harms native biodiversity. Here, we show the opposite to be true regarding the neglected and threatened guilds of epiphytes and lianas. We hope that our findings may help practitioners to reallocate scarce conservation resources away from management that harm native biodiversity, and provide them additional evidence of the benefits that pioneer species can bring for ecological restoration.

### Policy implications

Mauritius is globally well-known for some resounding conservation successes notably of birds (Sodhi et al., 2011) that were enabled by painstaking evidence-based approaches (Jones & Swinnerton, 1997; Swinnerton et al., 2004; Jones, 2008). More recently, however, some management of threatened species by conservation practitioners were applied that contradicted best available evidence (Florens, 2015; Florens, 2016), failed to reach the intended objectives and instead worsened the situation for threatened native biodiversity (Florens & Baider, 2019). A similar situation also emerged concerning ecological restoration for biodiversity conservation of the island’s most diverse native ecosystems whereby a policy of cutting back native plants was implemented. Indeed, following control of invasive alien plants, many native plants like woody lianas, strangler figs (FBV Florens, pers. obs., 2005 to present) and native pioneer trees like *H. madagascariensis* (Florens & Baider, 2013) that regenerated, were removed.

Mauritius was recently found to be the only place in the 12 or so million km^2^ of native range of *H. madagascariensis* where such a policy is applied (Baguette, Baider & Florens, 2025). By cutting back *H. madagascariensis*, the policy, at a cost, sets back restoration progress of the native woody plant community and increases the re-invasion by alien plants, hence the costs of maintenance weeding (Florens & Baider, 2013). While this policy has been supported and/or implemented by government conservation services and most of the main local conservation NGOs for over three decades, its proponents have in that time not yet been able to present evidence that it somehow benefits native or threatened biodiversity. In this context, we now present novel evidence that the policy of conservation managers cutting back native pioneer trees from areas undergoing ecological restoration, is in fact harmful to the native biodiversity of epiphytes and lianas in addition to the native trees themselves in addition to diverting scarce conservation resources elsewhere from where it is proven to be impactful, such as the control of invasive alien plants (Monty Florens & Baider, 2013; Krivek et al., 2020). We therefore recommend that the enduring practice of cutting or lopping branches of *H. madagascariensis* be stopped and be replaced instead by planting of the species where it can grow but where seed bank or natural germination is lacking. Some encouraging signs have started appearing with the first conservation organisation shifting its policy in 2025 from cutting to planting the species (Ferney Ltd., 2025).

## Conclusion

Using the widely distributed *Harungana madagascariensis* as a model, we show that pioneer trees, although relatively short-lived, can serve as important and even superior phorophytes for native epiphytes and lianas compared to the rest of the woody plant community where it grows. This finding was made within tropical forest areas undergoing ecological restoration following the weeding of invasive alien plants and was already apparent within the early stages after the weeding. This is good news for conservation in a place like Mauritius where much of the biota is highly threatened with extinction, and where epiphytes and lianas constitute a particularly diverse but largely overlooked component of native plant diversity despite the fact that they comprise many rare and threatened species, and ecologically important ones too, notably for some endemic threatened frugivorous birds. We show that pioneer trees can greatly support conservation of native epiphytes and lianas, and highlight concerns that local conservation efforts are being undermined when native pioneer species such as *H. madagascariensis* are actively removed from areas undergoing ecological restoration. A shift from the current hypothesis-based to an evidence-based conservation policy on that matter is therefore warranted, to promote faster and better ecological restoration, and optimize the use of conservation resources that in turn would enable a much needed upscaling of restoration efforts on the island.

## Supplemental Information

10.7717/peerj.20520/supp-1 Supplemental Information 1 Selection of epiphytes and lianas observed in Brise Fer and Mount Camizard

10.7717/peerj.20520/supp-2 Supplemental Information 2 List of potential phorophyte species selected to be included in the ’Same age’ phorophyte category Each species is listed with its average annual growth rate and growth rate ratio compared to *Harungana madagascariensis*, which has also been included for comparison.

10.7717/peerj.20520/supp-3 Supplemental Information 3 List of all epiphyte and liana species recorded under this study with their respective overall abundance

10.7717/peerj.20520/supp-4 Supplemental Information 4 List of all epiphyte and liana species recorded under this study with their respective abundance

10.7717/peerj.20520/supp-5 Supplemental Information 5 List of all epiphyte and liana species recorded under this study with their respective relative frequency and frequency of occurence

10.7717/peerj.20520/supp-6 Supplemental Information 6 List of epiphyte and liana families recorded under this study with their respective importance in terms of number of species and individuals recorded

10.7717/peerj.20520/supp-7 Supplemental Information 7 Raw phorophyte data collected under this study

10.7717/peerj.20520/supp-8 Supplemental Information 8 Raw epiphyte data collected under this study

## Additional Information and Declarations

### Data Availability

The following information was supplied regarding data availability: The data is available in the Supplementary Files.
