# Supplementary material for: Harungana madagascariensis (Hypericaceae) is a key phorophyte for native epiphytes and lianas during ecological restoration: case study on an oceanic island
Source: PeerJ. 2025 Dec 19;13:e20520. doi: 10.7717/peerj.20520 (PMC12721127; doi:10.7717/peerj.20520)
Supplement: Supplemental Information 1 [file peerj-13-20520-s001.pdf]

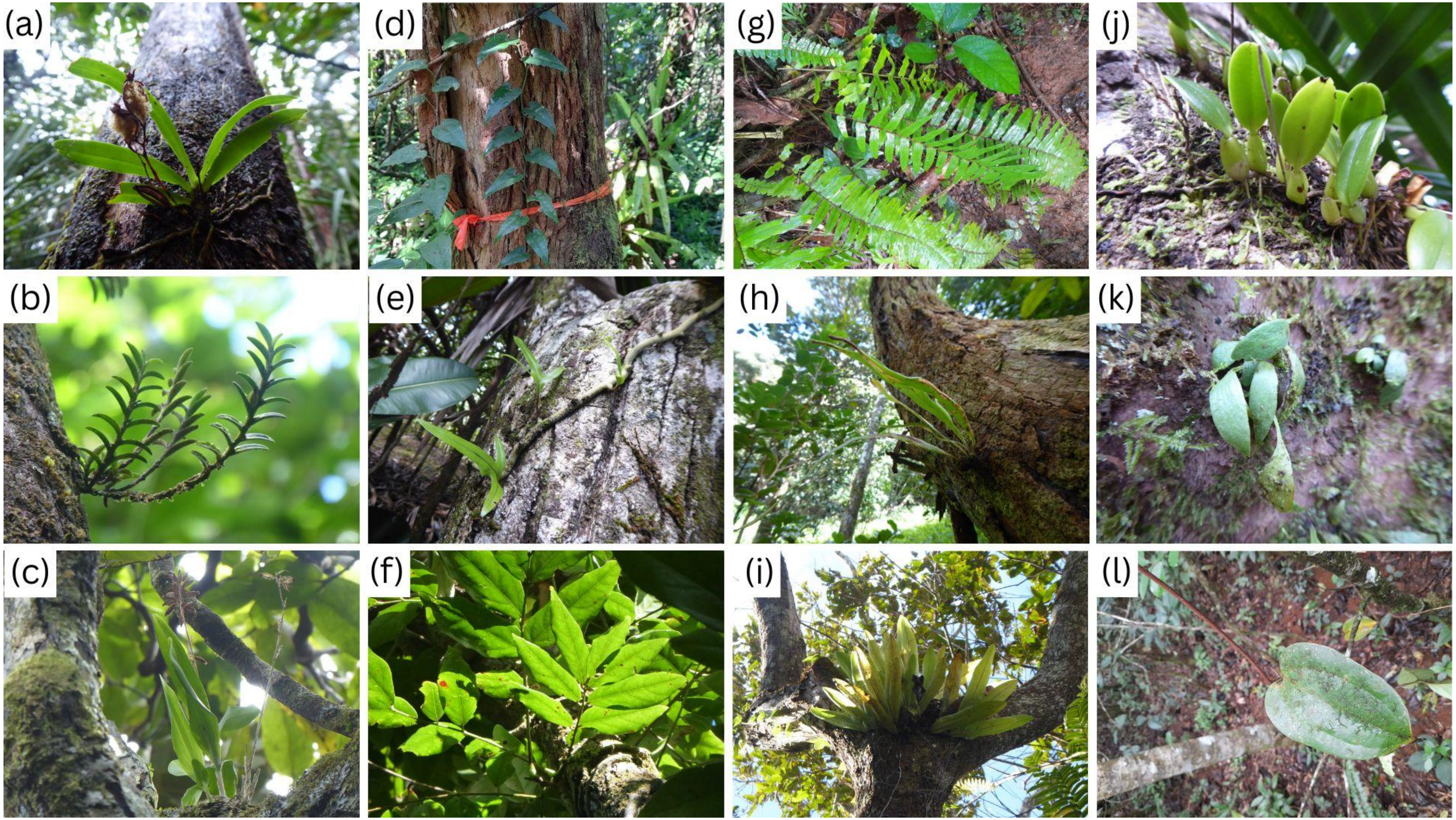

Selection of epiphytes and lianas observed in Brise Fer and Mount Camizard. (a) to (c) illustrate three of the most abundant orchid species observed, respectively *Angraecum* spp., *Angraecum pectinatum*, and *Polystachia mauritiana s.l.*. (b) to (f) illustrate three of the most abundant liana species observed, respectively *Piper borbonense*, *Microsorum scolopendria*, and *Cnestis polyphylla*. (g) to (i) illustrate three of the most abundant fern species observed, respectively *Nephrolepis biserrata*, *Lepisorus spicatus*, and *Microsorum punctatum*. (j) to (l) illustrate three of the rarest epiphyte and liana species observed, respectively *Bulbophyllum cf. incurvum*, Anthrophyum immersum, and Smilax anceps. Photos: François Baguette.
